# Association between early blood urea nitrogen-to-albumin ratio and one-year post-hospital mortality in critically ill surgical patients: a propensity score-matched study

**DOI:** 10.1186/s12871-023-02212-y

**Published:** 2023-07-21

**Authors:** Khoi Nguyen Nguyen, Tzu-I Chuang, Li-Ting Wong, Ming-Cheng Chan, Wen-Cheng Chao

**Affiliations:** 1 Division of Hepato-Biliary-Pancreatic Surgery, Chợ Rẫy Hospital, Ho Chi Minh, Vietnam; 2 Division of Chest Medicine, Department of Internal Medicine, Taichung Veterans General Hospital, Taichung, Taiwan; 3 Department of Medical Research, Taichung Veterans General Hospital, Taichung, Taiwan; 4 Department of Critical Care Medicine, Taichung Veterans General Hospital, Taichung, Taiwan; 5 Department of post-Baccalaureate Medicine, College of Medicine, National Chung Hsing University, Taichung, Taiwan; 6 Big Data Center, Chung Hsing University, Taichung, Taiwan; 7 Department of Automatic Control Engineering, Feng Chia University, Taichung, Taiwan; 8 Taichung Veterans General Hospital, No, 1650, Section 4, Taiwan Boulevard, Xitun District, Taichung City, 40705 Taiwan

**Keywords:** Blood urea nitrogen, Albumin, BAR, Long-term mortality, Surgical patients

## Abstract

**Background:**

Blood urea nitrogen to albumin ratio (BAR) is increasingly recognized as an early predictor for short-term outcomes in critically ill patients, but the association of BAR with long-term outcomes in critically ill surgical patients remains underexplored.

**Methods:**

We enrolled consecutive patients who were admitted to surgical intensive care units (ICUs) at Taichung Veterans General Hospital between 2015 and 2020, and the dates of death were retrieved from Taiwan’s National Health Insurance Research Database. In addition to Cox regression, we also used propensity score matching to determine the hazard ratios (HRs) and 95% confidence intervals (CIs) for one-year post-hospital mortality of the variables.

**Results:**

A total of 8,073 eligible subjects were included for analyses. We found that age, male gender, high Charlson Comorbidity Index, high Acute Physiology and Chronic Health Evaluation II score, positive microbial culture, and leukocytosis were predictors for mortality, whereas high body mass index, scheduled surgery, and high platelet counts were protective factors against long-term mortality. The high BAR was independently associated with increased post-hospital mortality after adjustment for the aforementioned covariates (adjHR 1.258, 95% CI, 1.127–1.405). Notably, the association tended to be stronger in females and patients with fewer comorbidities and lower disease severity of critical illness. The propensity score matching, dividing subjects by BAR higher or lower than 6, showed a consistent association between week-one BAR and post-hospital mortality (adjHR 1.503, 95% CI 1.247–1.811).

**Conclusions:**

BAR is a newly identified predictor of short-term outcome, and we identified long-term outcome-relevant factors, including BAR, and the identified factors may be useful for risk stratification of long-term outcomes in patients discharged from surgical ICUs.

**Supplementary Information:**

The online version contains supplementary material available at 10.1186/s12871-023-02212-y.

## Background

The long-term outcome of critically ill patients is an emerging issue in clinical practice; however, evidence focusing on critically ill surgical patients remains sparse [1–3]. Blood urea nitrogen (BUN) is a nitrogenous end-product of protein metabolism, and the elevated level of BUN reflects underlying catabolism in patients with acute illnesses, including acute myocardial infarction, pneumonia, acute pancreatitis, and infection with coronavirus disease 2019 (COVID-19) [4–8]. Albumin plays an essential physiological role, such as maintaining osmotic pressure as well as regulating fluid balance, and a low albumin level is a fundamental biomarker of protein-energy metabolism in critically ill surgical patients [9–11]. Increasing evidence shows the BUN to Albumin ratio (BAR) can predict the short-term outcome of critically ill patients [12–14]. For example, Dundar et al. found that an elevated BAR was a predictor of in-hospital mortality in older (> 65 years of age) emergency department patients [12]. A study conducted by Ata et al. found that BAR was a better independent predictor of in-hospital mortality than BUN or albumin alone in patients with severe COVID-19 infection [15]. Notably, Zhao D. et al. recently used the Medical Information Mart for Intensive Care III (MIMIC III) database with 1,462 patients who underwent coronary artery bypass grafting (CABG) and found that a high BAR during admission at the intensive care unit (ICU) was associated with long-term mortality [14]. These findings highlight the crucial role of BAR in critically ill surgical patients and indicate the need to clarify the prolonged impact of BAR on post-hospital mortality in critically ill surgical patients. In the present study, we linked data from the critical care database at Taichung Veterans General Hospital (TCVGH) with death registration data from Taiwan’s National Health Insurance Research Database to investigate factors associated with post-hospital long-term mortality and to determine the association between BAR and long-term mortality using both Cox regression and propensity score matching in critically ill surgical patients.

## Materials and methods

### Ethical approval

The study used de-identified data from the TCVGH critical care database and was approved by the Institutional Review Board of Taichung Veterans General Hospital (TCVGH: SE20249B-1), which waived the requirement for informed consent. The study was conducted in accordance with the Declaration of Helsinki.

### Subjects enrollment

The cohort study was conducted at TCVGH, a tertiary referral hospital with 1,530 beds in central Taiwan. We enrolled consecutive patients who were admitted to three surgical ICUs at TCVGH during 2015–2020. Given that one patient may be admitted to the ICU more than one time, we used data of the first ICU admission for analyses of patients with more than one ICU admission. We retrieved data from the critical care database at TCVGH to obtain information on variables related to critical care, including demographic data, comorbidities using the International Classification of Diseases, both 9th and 10th Revision, Clinical Modification (ICD-9/10-CM) codes, divisions of ICU admission, the Acute Physiology and Chronic Health Evaluation (APACHE) II score, administration of vasopressors, results of microbial culture, mechanical ventilation, and renal replacement therapy.

### Primary outcome

The primary outcome of interest of the present study was all-cause mortality after discharge from the surgical ICUs. To ascertain the date-of-death of enrolled subjects, we linked the aforementioned TCVGH critical care database with the death registration data of Taiwan’s National Health Insurance Research Database (NHIRD) [16]. Taiwan’s National Health Insurance (NHI) program is a single-payer, compulsory health insurance system that covers 99.9% of the population, and the information on date-of-death in this study was accurate.

### Statistical analyses

Descriptive data were presented as means ± standard deviation or number (percentages). Kaplan-Meier analysis was applied to illustrate the association between BAR and post-hospital mortality. The Cox proportional hazards model was used to determine hazard ratios (HRs) and 95% confidence intervals (CI) of risk factors for mortality after adjustment for age, sex, Charlson Comorbidity Index (CCI), APACHE II, presence of shock, fluid balance, positive microbial culture, anaemia, and potential cofounders, as performed in our previous studies [17–19]. Statistical analyses were two-sided, and the level of significance was 0.05. Data analyses were performed using R version 3.6.0.

### Propensity score matching

In the present study, we further utilized propensity score matching to mitigate potential selection bias and confounding effects between the two groups [20]. Propensity score matching (1:1) was performed to determine the impact of week-one BAR on one-year post-hospital mortality in critically ill surgical patients. We used the optimal nearest neighbor matching algorithm, and the caliper distance of the standard mean difference was 0.2 [21].

### Subgroup analyses

To determine the effect of modification of variables, we further used the Wald test to determine the significance of the modification effect by covariates, including age, sex, body mass index, comorbidities, and severities of critical illness [22].

## Results

### Demographic and data of the enrolled critically ill surgical patients

**Fig. 1:**
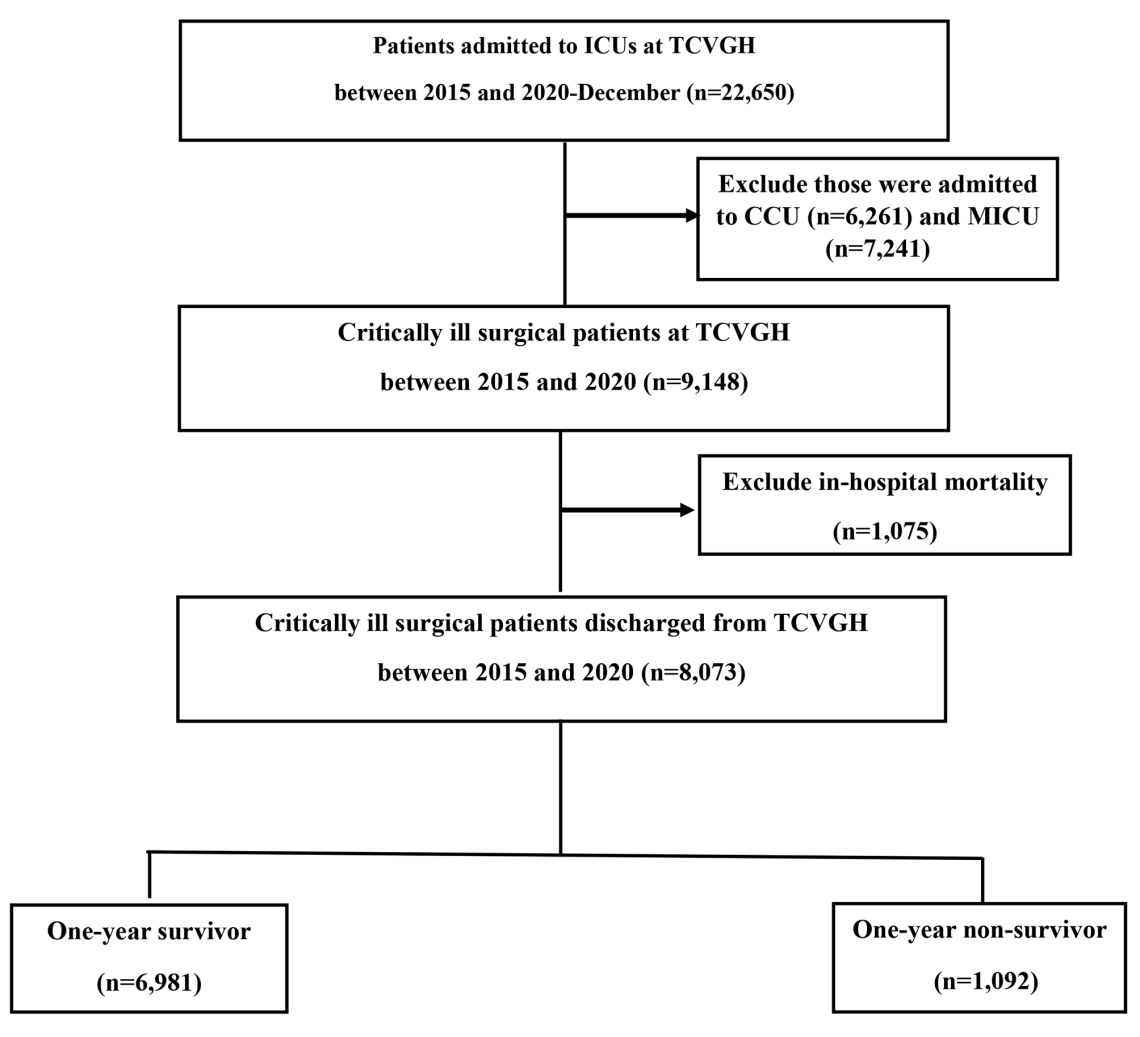
Flow chart of enrollment of subjects

In this study, we included 9,148 critically ill patients who were admitted to surgical ICUs at TCVGH between 2015 and 2020. We excluded 1,075 patients who died during hospitalization as the aim of this study was to identify factors associated with long-term, rather than short-term, outcomes. Of the 8,073 enrolled patients, 62.8% of them were male, and the average age was 60.4 ± 15.8 years (Fig. 1; Table 1). The average CCI was 1.6 ± 1.4, and the detailed comorbidities were summarised in the supplemental Table 1. The 30-day, 90-day, and one-year post-hospital mortality rates were 0.78% (63/8,073), 4.1% (329/8,073), and 13.5% (1,092/8,073), respectively. The main divisions of ICU admission were neurosurgery (51.6%), cardiovascular surgery (22.2%), and colorectal surgery/general surgery (10.6%). Among the enrolled subjects, 75.7% (6,111/8,073) of the patients had received the surgery during ICU admission, with 16.1% (1,297/8,073) and 59.6% (4,814/8,073) of the 6,111 patients receiving emergent surgery or scheduled surgery, respectively. Compared with the survivors, patients who died within one year after discharge appeared to have higher disease severities, with a higher APACHE II score (21.7 ± 5.3 vs. 19.8 ± 5.5, p < 0.001), were more likely to have shock (35.3% vs. 27.2%, p < 0.001), receive mechanical ventilation for more than 3 days (46.6% vs. 27.9%, p < 0.001), to undergo renal replacement therapy (6.8% vs. 3.1%, p < 0.001), to have a positive day 1–3 fluid balance (75.4% vs. 62.4%, p < 0.001), and to have a positive microbial culture (49.9% vs. 26.3%, p < 0.001). With regard to laboratory data, the non-survivors tended to have a lower level of hemoglobin (10.5 ± 1.7 vs. 11.5 ± 1.9 g/dL, p < 0.001), lower platelet counts (190.4 ± 94.1 vs. 203.0 ± 83.1 103/µL), and lower serum level of albumin (3.2 ± 0.7 vs. 3.6 ± 0.7 mg/dL, p < 0.001), but had higher levels of BUN (26.2 ± 19.0 vs. 19.4 ± 13.2, p < 0.001) and BAR (8.9 ± 7.4 vs. 5.8 ± 4.8, p < 0.001).

**Table 1:** Patient characteristics categorised by one-year post-hospital mortality in critically ill surgical patients

	All n = 8,073	Survivor n = 6,981	Non-survivor n = 1,092	*p* value
Basic characteristics				
Age, years	60.4 ± 15.8	59.4 ± 15.7	67.2 ± 14.7	< 0.001
Male	5073 (62.8%)	4314 (61.8%)	759 (69.5%)	< 0.001
Body mass index	24.3 ± 4.4	24.5 ± 4.4	22.7 ± 4.2	< 0.001
Charlson Comorbidity Index	1.6 ± 1.4	1.5 ± 1.4	2.2 ± 1.5	< 0.001
**Severity of critical illness**				
APACHE II score	20 ± 5.5	19.8 ± 5.5	21.7 ± 5.3	< 0.001
Presence of shock	2288 (28.3%)	1902 (27.2%)	386 (35.3%)	< 0.001
**Surgical divisions**				< 0.001
Cardiovascular surgery	1794 (22.2%)	1673 (24.0%)	121 (11.1%)	< 0.001
Neurosurgery	4169 (51.6%)	3778 (54.1%)	391 (35.8%)	
Major abdomen surgery	854 (10.6%)	616 (8.8%)	238 (21.8%)	
Others	1256 (15.6%)	914 (13.1%)	342 (31.3%)	
**Managements in ICU**				
Emergent surgery	1297 (16.1%)	1144 (16.4%)	153 (14%)	0.052
Scheduled surgery	4814 (59.6%)	4233 (60.6%)	581 (53.2%)	< 0.001
Mechanical ventilation (> 3 days)	2454 (30.4%)	1945 (27.9%)	509 (46.6%)	< 0.001
Receiving RRT	290 (3.6%)	216 (3.1%)	74 (6.8%)	< 0.001
Fluid balance day 1–3, positive	5987 (65.5%)	4354 (62.4%)	1633 (75.4%)	< 0.001
Positive microbiological culture	2379 (29.5%)	1834 (26.3%)	545 (49.9%)	< 0.001
**Laboratory data**				
White blood cell count (/µl)	10,697 ± 4022	10643.1 ± 3685.5	11041.9 ± 5713.5	0.026
Haemoglobin (g/dL)	11.4 ± 1.9	11.5 ± 1.9	10.5 ± 1.7	< 0.001
Platelet (10^3^/µL)	201.3 ± 84.8	203.0 ± 83.1	190.4 ± 94.1	< 0.001
Creatinine (mg/dL)	1.2 ± 1.3	1.1 ± 1.3	1.4 ± 1.5	< 0.001
BUN (mg/dL)	20.3 ± 14.3	19.4 ± 13.2	26.2 ± 19.0	< 0.001
Albumin (mg/dL)	3.6 ± 0.7	3.6 ± 0.7	3.2 ± 0.7	< 0.001
Ratio of BUN/Albumin	6.2 ± 5.3	5.8 ± 4.8	8.9 ± 7.4	< 0.001
**Outcomes**				
ICU length of stay, days	6.4 ± 4.8	5.8 ± 4.5	8.1 ± 5.0	< 0.001
Hospital length of stay, days	17.0 ± 9.7	16.3 ± 9.3	19.1 ± 10.7	< 0.001
**Mortality at distinct time points**				
30-day mortality	63 (0.78%)	NA	63 (5.8%)	NA
90-day mortality	329 (4.1%)	NA	329 (30.1%)	NA

Abbreviations: APACHE, acute physiology and chronic health evaluation; ICU, intensive care unit; RRT, renal replacement therapy; BUN, Blood urea nitrogen

### Association between week-one BUN/Albumin ratio and post-hospital one-year mortality

**Fig. 2:**
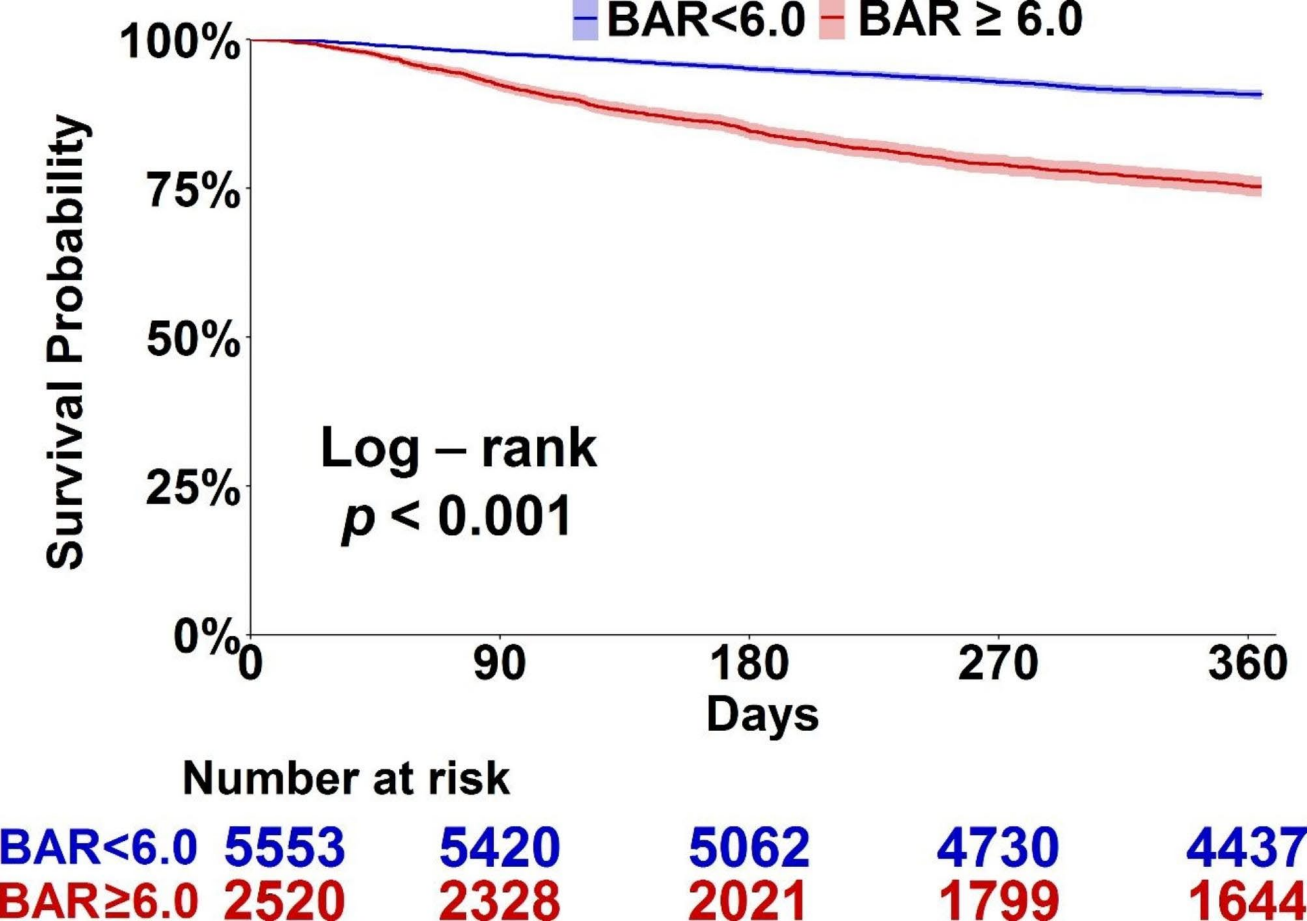
Kaplan–Meier survival curves stratified by median level of BUN/Albumin ratio higher or lower than 6.0 (Abbreviations: BUN, blood urea nitrogen)

We used the Kaplan-Meier plot and BAR 6.0, the integral level of the median of BAR among enrolled subjects, as the cut-point value to illustrate that a high BAR was associated with increased post-hospital mortality (Fig. 2). The multivariable Cox proportional hazards model was constructed to identify factors associated with one-year post-hospital mortality in critically ill surgical patients. We found that older age (adjHR 1.253, 95% CI 1.104–1.422), male gender (adjHR 1.379, 95% CI 1.210–1.572), high CCI (adjHR 2.200, 95% CI 1.929–2.508), increased APACHE II score (adjHR 1.016, 95% CI 1.004–1.029), positive microbial culture (adjHR 1.577, 95% CI 1.359–1.829), and leukocytosis (adjHR 1.024, 95% CI 1.010–1.037) were predictors for mortality, whereas high BMI (adjHR 0.919, 95% CI 0.905–0.933), scheduled surgery (adjHR 0.858, 95% CI 0.757–0.973), and high platelet counts (adjHR 0.831, 95% CI 0.798–0.867) were protective factors against long-term mortality. Notably, the high BAR was independently correlated with increased post-hospital mortality after adjustment for the aforementioned covariates (adjHR 1.258, 95% CI, 1.127–1.405) (Table 2). We further assessed the variables with a potential to modify the association between BAR and long-term mortality and found that the correlation tended to be stronger in females and patients with fewer comorbidities and lower disease severity. The association was higher in patients with CCI < 2, APACHE II < 25, no need for hemodialysis, negative microbial culture, level of hemoglobin higher than 10 g/dL, and serum level of creatinine less than 1.5 mg/dL (Table 3). Taken together, we found that high BAR was independently correlated with post-hospital mortality in critically ill surgical patients and the correlation was stronger in patients with fewer comorbidities and low severity of critical illness.

**Table 2:** Cox proportional hazard regression analysis for post-hospital mortality in critically ill surgical patients

	Univariable analysis		Multivariable analysis	
	HR (95% CI)	p value	HR (95% CI)	P value
Basic characteristics				
Age ≥ 65 years	1.880 (1.668–2.118)	< 0.001	1.253 (1.104–1.422)	< 0.001
Sex (Female)	1.367 (1.202–1.555)	< 0.001	1.379 (1.210–1.572)	< 0.001
Body mass index	0.903 (0.890–0.917)	< 0.001	0.919 (0.905–0.933)	< 0.001
Charlson comorbidity index ≥ 2	2.787 (2.460–3.156)	< 0.001	2.200 (1.929–2.508)	< 0.001
**Severity and managements**				
APACHE II score	1.069 (1.057–1.082)	< 0.001	1.016 (1.004–1.029)	0.008
Presence of shock	1.441 (1.273–1.631)	< 0.001	0.936 (0.819–1.070)	0.330
Scheduled surgery	0.736 (0.653–0.828)	< 0.001	0.858 (0.757–0.973)	0.017
Mechanical ventilation (≥ 3 days)	2.116 (1.879–2.383)	< 0.001	1.061 (0.911–1.235)	0.446
Fluid balance day 1–3, per liter increment	1.081 (1.054–1.108)	< 0.001	0.992 (0.966–1.018)	0.523
Positive microbiological culture	2.610 (2.318–2.938)	< 0.001	1.577 (1.359–1.829)	< 0.001
**Laboratory data**				
WBC counts, per 10^3^/µL increment	1.025 (1.011–1.038)	< 0.001	1.024 (1.010–1.037)	< 0.001
Haemoglobin, per 1 g/dL increment	0.998 (0.998–0.999)	< 0.001	1.000 (0.999-1.000)	0.533
Platelet, per 10^3^/µL increment	0.728 (0.702–0.755)	< 0.001	0.831 (0.798–0.867)	< 0.001
Creatinine, higher than 1.5 mg/dL	2.133 (1.856–2.452)	< 0.001	0.831 (0.682–1.013)	0.066
**BUN/Albumin ratio**, per 10 increment	1.803 (1.695–1.918)	< 0.001	1.258 (1.127–1.405)	< 0.001

Abbreviations: HR, hazard ratio; CI, confidence interval; APACHE IV, acute physiology and chronic health evaluation IV; WBC, white blood cell; BUN, blood urea nitrogen

**Table 3:** Effect modification of variables on the association between week-one ratio of BUN/Albumin and risk of post-hospital mortality in critically ill surgical patients

Variables	Subgroup	Crude HR (95%CI)	p for interaction	Adjusted HR (95%CI)	p for interaction
**Age**	< 65	1.750 (1.585–1.933)	0.792	1.505 (1.342–1.688)	0.982
	≥ 65	1.711 (1.567–1.869)		1.615 (1.474–1.770)	
**Sex**	Female	2.105 (1.908–2.321)	< 0.001	1.696 (1.514-1.900)	0.004
	Male	1.648 (1.519–1.788)		1.482 (1.352–1.624)	
**Body mass index**	< 21.5	1.671 (1.527–1.829)	0.115	1.479 (1.333–1.642)	0.302
	≥ 21.5	1.853 (1.702–2.017)		1.580 (1.435–1.740)	
**CCI**	< 2	1.988 (1.780–2.220)	< 0.001	1.838 (1.617–2.090)	< 0.001
	≥ 2	1.489 (1.372–1.617)		1.478 (1.360–1.607)	
**APACHE II score**	< 25	1.905 (1.771–2.049)	< 0.001	1.675 (1.538–1.825)	< 0.001
	≥ 25	1.312 (1.156–1.489)		1.202 (1.052–1.374)	
**Presence of shock**	No	1.868 (1.718–2.031)	0.083	1.568 (1.424–1.727)	0.421
	Yes	1.653 (1.498–1.825)		1.523 (1.366–1.699)	
**Surgical divisions**	CVS	2.623 (2.213–3.109)	0.927	2.391 (1.990–2.873)	0.064
	Neurosurgery	2.447 (1.987–3.015)		1.826 (1.435–2.325)	
	Abdomen surgery	1.400 (1.127–1.739)		1.287 (1.027–1.611)	
**Hemodialysis**	No	2.189 (2.018–2.373)	< 0.001	1.832 (1.672–2.008)	< 0.001
	Yes	1.214 (1.014–1.454)		1.226 (1.020–1.474)	
**Fluid balance, day 1–3**	Negative	2.068 (1.841–2.321)	0.010	1.707 (1.495–1.949)	0.108
	Positive	1.701 (1.579–1.833)		1.511 (1.388–1.644)	
**Positive microbiological culture**	No	2.045 (1.832–2.284)	< 0.001	1.679 (1.478–1.906)	< 0.001
	Yes	1.427 (1.308–1.556)		1.281 (1.164–1.410)	
**White blood cell count** (/µl)	< 12,000	1.875 (1.734–2.027)	0.096	1.621 (1.480–1.775)	0.113
	≥ 12,000	1.673 (1.511–1.853)		1.443 (1.283–1.622)	
**Haemoglobin** (g/dL)	< 10	1.322 (1.207–1.448)	< 0.001	1.201 (1.088–1.327)	< 0.001
	≥ 10	2.387 (2.118–2.690)		1.926 (1.678–2.211)	
**Platelet** (103/µL)	< 150	1.594 (1.447–1.756)	0.003	1.483 (1.335–1.647)	0.094
	≥ 150	1.943 (1.781–2.119)		1.617 (1.461–1.789)	
**Creatinine** (mg/dL)	< 1.5	3.656 (3.176–4.208)	< 0.001	3.236 (2.730–3.835)	< 0.001
	≥ 1.5	1.380 (1.228–1.550)		1.353 (1.199–1.527)	

Abbreviations: BUN, blood urea nitrogen; HR, hazard ratio; CI, confidence interval; CCI, Charlson comorbidity index; APACHE IV, acute physiology and chronic health evaluation IV; CVS, cardiovascular surgery

### Propensity-matched approach to verify the association between week-one BAR and one-year post-hospital mortality in critically ill surgical patients

In addition to the Cox regression model, we further used the propensity score-based approach to verify the aforementioned association between week-one BAR and one-year mortality in critically ill surgical patients. To be consistent with the Kaplan-Meier plot (Fig. 2), we also divided the enrolled subjects by BAR 6.0 and then used 1:1 propensity score matching for the following analyses (Supplemental Fig. 1 for the flow diagram of propensity score matching). Supplemental Table 1 summarizes the characteristics of the selected subjects whose BAR values were higher and lower than 6.0 (Supplemental Table 2 for characteristis between the patients in the primary cohort and propensity score-matched cohort). The quality of matching was high as the standardized difference of covariates was lower than 0.15 (Supplemental Fig. 2 for the standardised mean difference in primary and propensity score-matched populations). We found a consistent association between week-one BAR and post-hospital mortality in both the primary cohort and PSM cohort, with an adjHR in the PSM of 1.503 (95% CI 1.247–1.811) (Table 4).

**Table 4:** Cox proportional hazard regressions for estimation of the association between ration of BUN/albumin higher than 6.0 and post-hospital mortality in critically ill surgical patients

	Primary cohort		PSM cohort	
	HR (95%CI)		HR (95%CI)	
**Model 1**	2.949 (2.618–3.323)	< 0.001	2.949 (2.618–3.323)	< 0.001
**Model 2**	1.846 (1.614–2.111)	< 0.001	1.527 (1.268–1.839)	< 0.001
**Model 3**	1.588 (1.366–1.845)	< 0.001	1.503 (1.247–1.811)	< 0.001

Model 1. Unadjusted

Model 2. Adjusted for demographic data, severity and managements

Model 3. Adjusted for variables in model 2 and Laboratory parameters

Abbreviations: BUN, blood urea nitrogen; PSM, propensity score matching; HR, hazard ratio; CI, confidence interval

## Discussion

In this study, we investigated the association between early BAR and long-term post-hospital outcome in critically ill surgical patients. We found that high week-one BAR correlated with post-hospital mortality in critically ill surgical patients, and the association tended to be stronger in females and patients with fewer comorbidities and lower disease severity of critical illness. In addition to the aforementioned Cox regression model, we also used a propensity score-matched approach to demonstrate a consistent association between week-one BAR and post-hospital mortality in critically ill surgical patients. These data highlight the previously underexplored long-term outcome-relevant factors that may be used for risk stratification of long-term outcomes in patients discharged from surgical ICUs.

Long-term outcomes are increasingly recognized as an essential issue in critical care medicine, but it is somewhat difficult to delineate the impact of management in critical illness on short-term and long-term outcomes [1]. The majority of studies that explored early determinants of long-term outcomes used long-term survival as the independent variable; however, long-term survival without the exclusion of hospital mortality might mainly reflect the severity of critical illness rather than the prolonged impact of critical illness [23]. Moreover, most studies exploring long-term outcomes were conducted in medical/mixed ICUs, whereas studies focusing on critically ill surgical patients are sparse [2]. In the present study, we excluded those who died during admission, and the findings support the prognostic role of week-one BAR among patients discharged from surgical ICUs.

BUN is a low-molecular-weight nitrogen product derived from protein catabolism and excreted by the kidney [24]. Therefore, BUN reflects protein intake, endogenous protein catabolism, fluid balance, hepatic urea synthesis, and fluid/renal status in critically ill patients and have been widely used as a prognostic marker in patients with various surgical diseases [5, 7, 25]. Similarly, albumin also has various biological effects, including maintenance of osmotic pressure, binding and transport of various drugs, and neutralization of free radicals, and hypoalbuminemia has been identified as a poor prognostic factor in critically ill surgical patients [9, 11, 26]. Given that BAR, consisting of elevated BUN with hypoalbuminemia, appeared to be involved in the aforementioned physiological mechanisms, accumulating evidence indicates that high BAR was an independent poor prognostic factor in a variety of diseases, including ischemic stroke, community-acquired pneumonia, gastrointestinal bleeding, and COVID-19 infection [13, 27–29].

In the present study, we provide further clinical evidence on the long-term mortality impact of high BAR in the first week in critically ill surgical patients. We postulate that the high BAR may reflect not only organ dysfunction but also ongoing catabolism with impaired protein/albumin synthesis, which is a persistent catabolic state after critical illness [30, 31]. Recent evidence further suggests that hypercatabolism occurs along with persistent inflammation and immunosuppression, giving rise to a syndrome termed PICS (persistent inflammation, immunosuppression and catabolism syndrome). A number of therapeutic approaches, including specific nutritional support, anti-inflammatory agents, anabolic agents, antioxidants, microbiota modulators, and early mobilization, are currently being explored with a view to ameliorating hypercatabolism in critically ill patients [31, 32]. Intriguingly, we not only found that high week-one BAR was correlated with increased post-hospital mortality, but also noted that the aforementioned correlation tends to be stronger among those with relatively fewer comorbidities or low severities of critical illness (Table 3). It is reasonable to suppose that dysregulated protein metabolism and nutritional status would be more likely to be restored within one week in critically ill surgical patients with low disease severity compared with those with high disease severity.

Recently, Zhao D et al. used Medical Information Mart for Intensive Care (MIMIC) III databases to analyze 1,462 patients receiving CABG, and found that BAR higher than 6.45 was associated with increased long-term mortality, which was consistent with our findings [14]. In their study, Zhao D et al. found a higher BAR was an independent risk factor of one-year mortality (HR 3.904; 95% CI 2.559–5.956) [14]. In the present study, we excluded patients who died during admission and focused on the 8,073 patients discharged from surgical ICUs. Furthermore, we found a consistent association between BAR and post-hospital mortality among those receiving cardiovascular surgery, neurosurgery, and abdominal surgery (Table 3). Moreover, we used propensity score matching to further validate the association between BAR and post-hospital mortality in addition to the Cox regression model. Additionally, whereas the MIMIC III (2001–2012) covers a relatively old time span, the dataset used in this study represents the current (2015–2020) management and prognosis of critically ill surgical patients. Both the present study and the study conducted by Zhao D et al. were single-center studies, and therefore, prospective multi-center studies are warranted to confirm our findings.

We used BAR 6.0 as the cut-point value in this study to achieve the balance between patients with high and low BAR. A number of BAR relevant studies have used distinct cut-point values [12–14, 28, 33], and we hence tested the consistence of our results using distinct cut-point values of BAR. We found a consistent association between week-one BAR and one-year post-hospital mortality among the enrolled critically ill surgical patients (Supplemental Fig. 3).

There were limitations in this study. First, the study was an observational study, and therefore, it was only possible to observe associations, rather than causal effects of BAR on mortality. Second, multi-center studies are needed to validate our findings, even though the number of subjects in the present study was high. Third, the potential unmeasured confounders in this retrospective study remain a concern despite the application of propensity score matching. Additionally, the potential existence of some unmeasured confounders, such as stroke, coronary arterial disease, or autoimmune disease.

## Conclusion

Long-term outcome-associated early predictors are an emerging research priority in critically ill surgical patients, and recent studies have shown that BAR was a predictor of short-term outcome in patients with inflammatory diseases and critical illnesses. In the present study, we linked two databases to investigate the factors associated with one-year post-hospital mortality in critically ill surgical patients. We used both the Cox regression model and propensity score matching approach to identify a consistent association between week-one BAR and one-year post-hospital mortality in critically ill surgical patients. Our findings highlight the crucial role of BAR as a long-term prognostic factor in critically ill surgical patients, and more studies are warranted to elucidate the underlying mechanism.

## Electronic supplementary material

Below is the link to the electronic supplementary material.

Supplementary Material 1

## Data Availability

The data underlying this article will be shared upon reasonable request to the corresponding author.
