# Supplementary material for: Association between early blood urea nitrogen-to-albumin ratio and one-year post-hospital mortality in critically ill surgical patients: a propensity score-matched study
Source: BMC Anesthesiol. 2023 Jul 21;23:247. doi: 10.1186/s12871-023-02212-y (PMC10362554; doi:10.1186/s12871-023-02212-y)
Supplement: Supplementary file 1 — Supplementary Material 1 [file 12871_2023_2212_MOESM1_ESM.pdf]

Supplemental Table 1. Detailed comorbidities of enrolled critically ill surgical patients

	All<br>n=8,073	Survivor<br>n=6,981	Non-survivor<br>n=1,092	*p* value
**Charlson Comorbidity Index**	1.6±1.4	1.5±1.4	2.2±1.5	<0.001
Acute myocardial infarction	975 (12.1%)	884 (12.7%)	91 (8.3%)	<0.001
Congestive heart failure	495 (6.1%)	419 (6%)	76 (7%)	0.247
Peripheral vascular disease	684 (8.5%)	582 (8.3%)	102 (9.3%)	0.294
Cerebrovascular disease	2244 (27.8%)	2017 (28.9%)	227 (20.8%)	<0.001
Dementia	165 (2%)	116 (1.7%)	49 (4.5%)	<0.001
Asthma	150 (1.9%)	116 (1.7%)	34 (3.1%)	0.001
Chronic pulmonary disease	121 (1.5%)	106 (1.5%)	15 (1.4%)	0.816
Rheumatic disease	127 (1.6%)	110 (1.6%)	17 (1.6%)	0.933
Peptic ulcer disease	397 (4.9%)	328 (4.7%)	69 (6.3%)	0.026
Mild liver disease	435 (5.4%)	361 (5.2%)	74 (6.8%)	0.035
Diabetes	1642 (20.3%)	1386 (19.9%)	256 (23.4%)	0.007
Hemiplegia or paraplegia	155 (1.9%)	137 (2%)	18 (1.6%)	0.559
Renal disease	769 (9.5%)	591 (8.5%)	178 (16.3%)	<0.001
Malignant tumors	1849 (22.9%)	1268 (18.2%)	581 (53.2%)	<0.001
Solid metastatic tumor	1810 (22.4%)	1240 (17.8%)	570 (52.2%)	<0.001
Lymphoma and leukemia	91 (1.1%)	60 (0.9%)	31 (2.8%)	<0.001
Metastatic tumor	795 (9.8%)	430 (6.2%)	365 (33.4%)	<0.001
Moderate or severe liver disease	121 (1.5%)	105 (1.5%)	16 (1.5%)	0.972
End-stage renal disease	237 (2.9%)	178 (2.5%)	59 (5.4%)	<0.001

Supplemental Table 2. Characteristics between the patients categorised by BUN/Albumin ratio in the primary cohort and propensity score-matched cohort

Basic characteristics	Before PSM			1:1 PSM		
	BUN/Albumin ratio <6.0 (n = 5,553)	BUN/Albumin ratio ≥6.0 (n = 2,520)	p value	BUN/Albumin ratio <6.0 (n = 1,264)	BUN/Albumin ratio ≥6.0 (n = 1,264)	p value
**Demographic data**						
**Age ≥65 years**	1801 (32.4%)	1573 (62.4%)	<0.001	694 (54.9%)	712 (56.3%)	0.496
**Sex (Female)**	3367 (60.6%)	1706 (67.7%)	<0.001	852 (67.4%)	852 (67.4%)	1.000
**BMI ≥ 27**	24.3±4.4	24.3±4.6	0.842	24.0±4.3	24.1±4.5	0.486
**CCI ≥ 2**	1908 (34.4%)	1522 (60.4%)	<0.001	649 (51.3%)	649 (51.3%)	1.000
**Severities and managements**						
**APACHE II score**	19.2±5.4	21.8±5.2	<0.001	20.4±5.1	20.8±5.1	0.084
**Mechanical ventilation**	1231 (22.2%)	1223 (48.5%)	<0.001	510 (40.3%)	510 (40.3%)	1.000
**Presence of shock**	1220 (22.0%)	1068 (42.4%)	<0.001	415 (32.8%)	415 (32.8%)	1.000
**Scheduled surgery**	3496 (63.0%)	1318 (52.3%)	<0.001	695 (55.0%)	695 (55.0%)	1.000
**Fluid balance day 1-3, liter**	0.4±1.8	1.3±2.7	<0.001	1.0±2.0	1.0±2.5	0.461
**Culture positivity**	1183 (21.3%)	1196 (47.5%)	<0.001	499 (39.5%)	499 (39.5%)	1.000
**Laboratory parameters**						
**White blood cell, ≥12000/µL**	1572 (28.3%)	884 (35.1%)	<0.001	425 (33.6%)	441 (34.9%)	0.530
**Hemoglobin, g/dL**	11.9±1.9	10.3±1.6	<0.001	10.9±1.6	10.8±1.7	0.365
**Platelet, 10^3/µL**	214.5±80.1	172.4±87.6	<0.001	189±78.8	185.9±92	0.333
**Creatinine, ≥1.5 mg/dL**	100 (1.8%)	994 (39.4%)	<0.001	91 (7.2%)	91 (7.2%)	1.000

Abbreviations: BUN, blood urea nitrogen; PSM, propensity score matching; BMI, body mass index; CCI, Charlson comorbidity index; APACHE IV, acute physiology and chronic health evaluation IV.

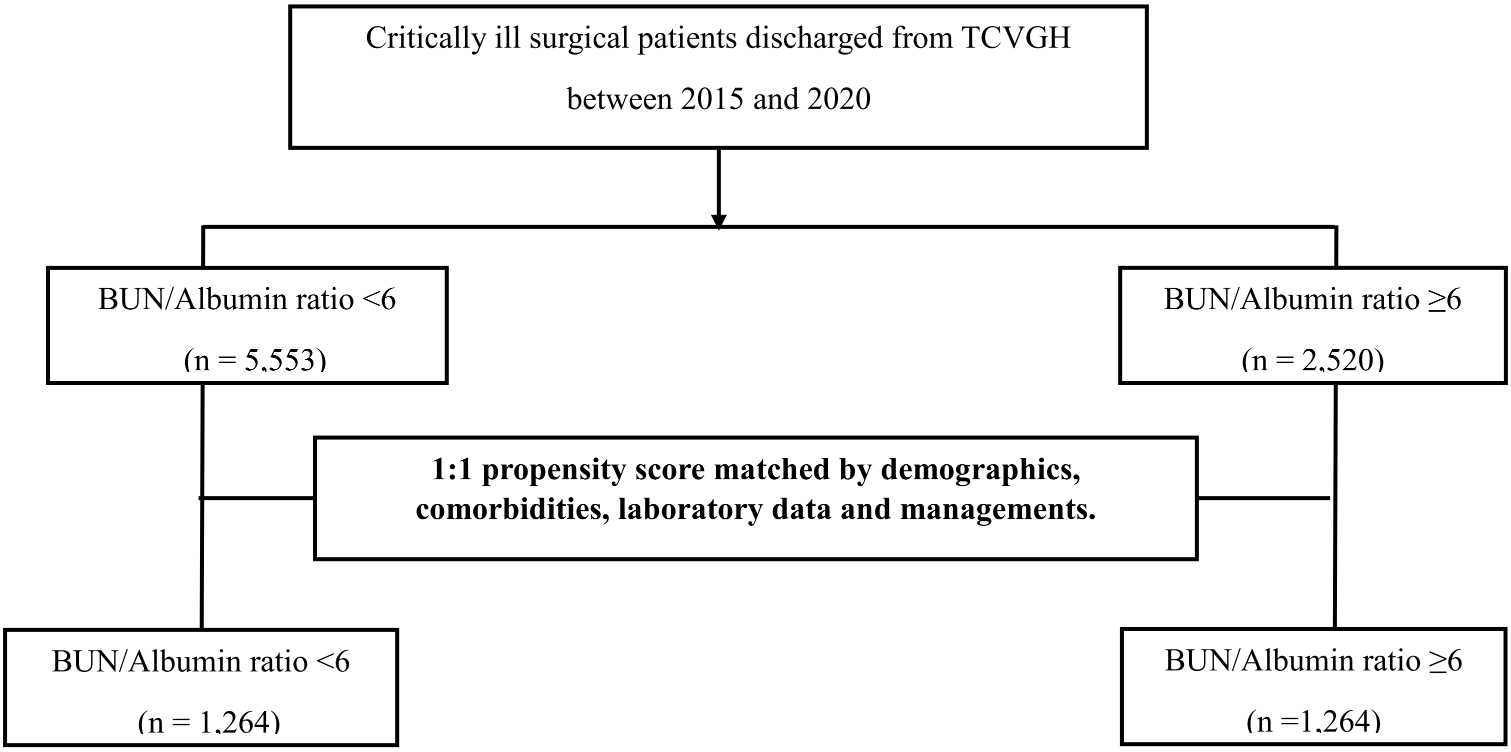

Supplemental Figure 1. Flowchart of propensity matching. Abbreviations: TCVGH, Taichung Veterans General Hospital; BUN, blood urea nitrogen.

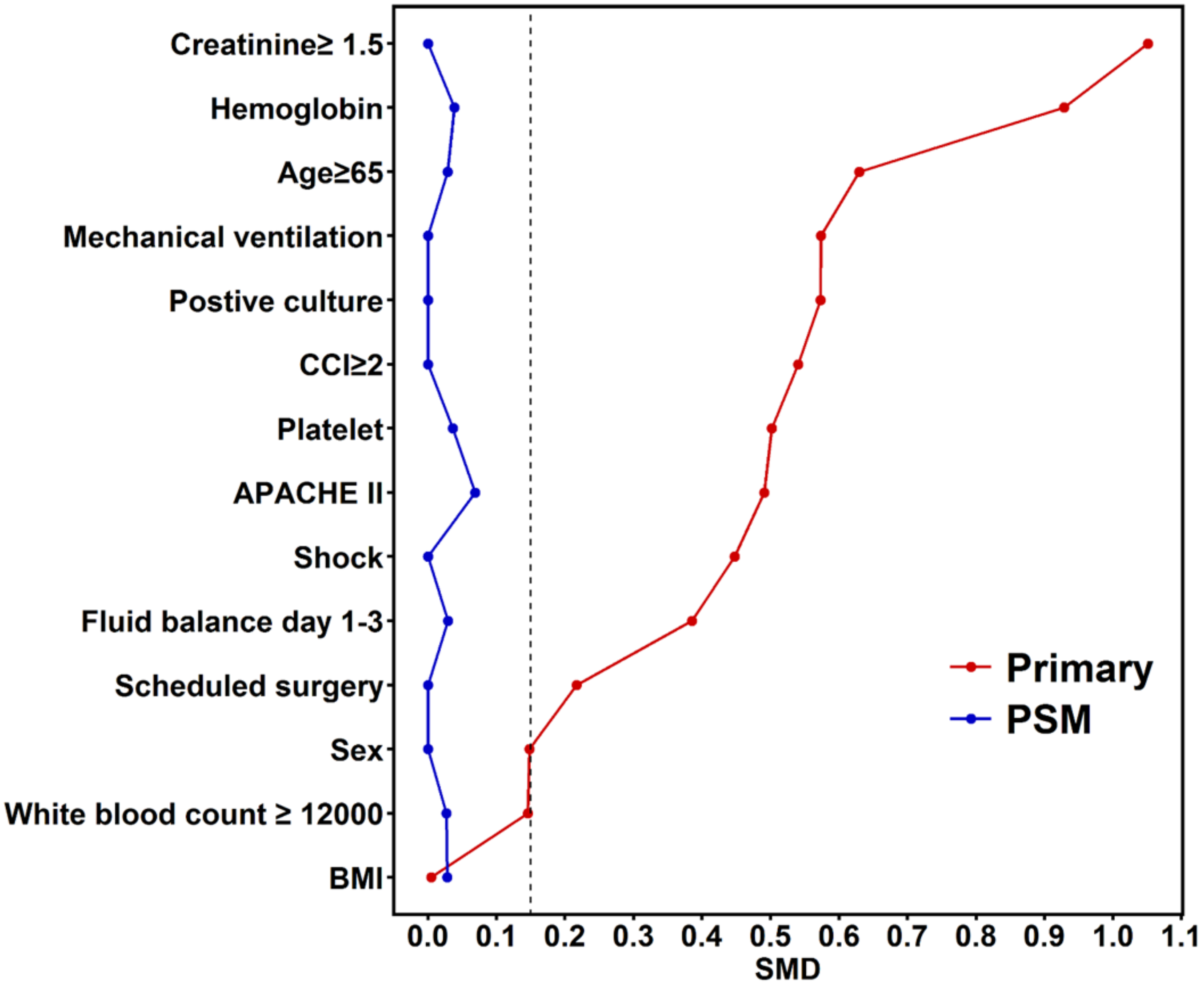

Supplemental Figure 2. Standardised mean difference between the patients whose ratio of BUN/Albumin higher and lower than 6.0 in primary and propensity score-matched cohort. Variables were ranked by the SMD. Abbreviations: SMD, standard mean difference; CCI, Charlson comorbidity index; APACHE II, acute physiology and chronic health evaluation II; BMI, body mass index.

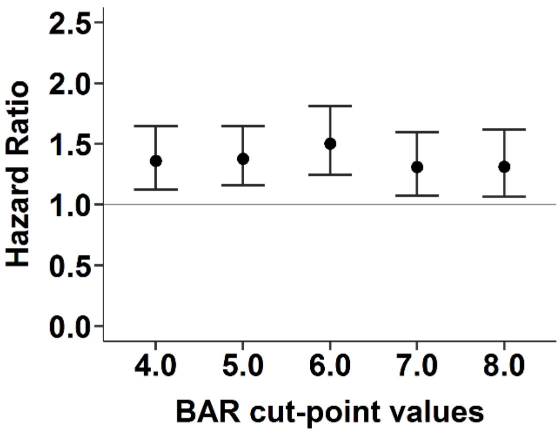

Supplemental Figure 3. The association between week-one BAR, using distinct cut-point values, and post-hospital mortality in PSM analyses. (BAR, blood urea nitrogen to albumin ratio; PSM, propensity score-matching)
